# Kinetic and mechanistic analysis of NH_3_ decomposition on Ru(0001), Ru(111) and Ir(111) surfaces†

**DOI:** 10.1039/d1na00015b

**Published:** 2021-02-09

**Authors:** Xiuyuan Lu, Jing Zhang, Wen-Kai Chen, Alberto Roldan

**Affiliations:** Cardiff Catalysis Institute, School of Chemistry, Cardiff University Main Building, Park Place Cardiff CF10 3AT UK roldanmartineza@cardiff.ac.uk; College of Chemistry, Fuzhou University Fuzhou Fujian 350116 China

## Abstract

We investigated the catalytic NH_3_ decomposition on Ru and Ir metal surfaces using density functional theory. The reaction mechanisms were unraveled on both metals, considering that, on the nano-scale, Ru particles may also present an fcc structure, hence, leading to three energy profiles. We implemented thermodynamic and kinetic parameters obtained from DFT into microkinetic simulations. Batch reactor simulations suggest that hydrogen generation starts at 400 K, 425 K and 600 K on Ru(111), Ru(0001) and Ir(111) surfaces, respectively, in excellent agreement with experiments. During the reaction, the main surface species on Ru are NH, N and H, whereas on Ir(111), it is mainly NH. The rate-determining step for all surfaces is the formation of molecular nitrogen. We also performed temperature-programmed reaction simulations and inspected the desorption spectra of N_2_ and H_2_ as a function of temperature, which highlighted the importance of N coverage on the desorption rate.

## Abstract 2

The NH_3_ decomposition mechanisms on Ru, hcp and fcc, and Ir were unraveled by DFT and implemented in microkinetic simulations resulting in good agreement with experiments.

## Graphical abstract

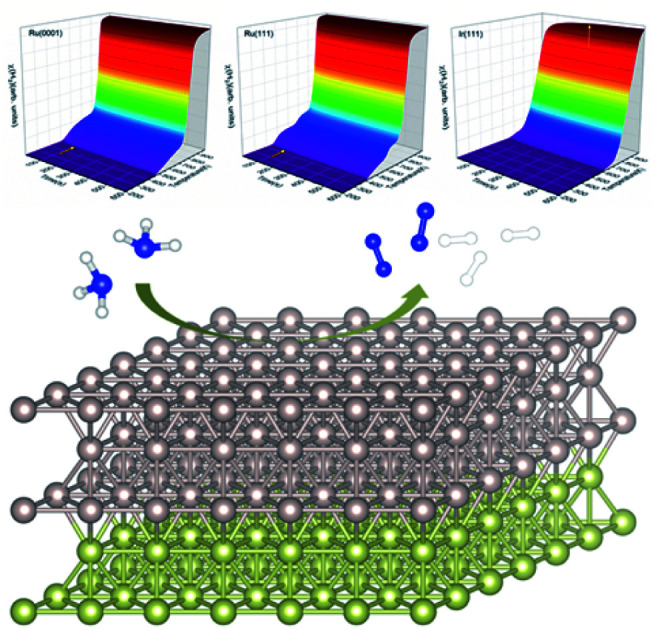

## 1. Introduction

Current environmental concerns are drawing the attention of all communities to exploit resources with low or even zero carbon emission.^1^ Molecular hydrogen is recognized as an energy vector to drive sustainable growth; nevertheless, it presents high risks and cost associated with its transport and storage.^2^ Alternatively, ammonia (NH_3_) is a suitable carbon-free molecule to store H_2_, as its decomposition produces only H_2_ and N_2_.^3,4^ It is easy to transport and store as it is liquid at room temperature under low pressure. Every year, around 150 million tons of NH_3_ is synthesized and traded around the world.^5^ Indeed, the high hydrogen content of NH_3_ (17.64 wt%) makes it more attractive than bulk commodities such as methanol (12.50 wt%), ethanol (13.04 wt%), formic acid (4.35 wt%) and acetic acid (6.66 wt%). Although the decomposition of NH_3_ is an endothermic process, the oxidation of the produced H_2_ (as fuels) is highly exothermic, making this reaction worthwhile.^6^ The presence of a catalyst can facilitate the NH_3_ decomposition, and therefore, detailed investigations on mechanisms and their limitations are of paramount importance to develop selective and efficient catalysts.

Extensive studies have shed light on the ammonia decomposition mechanisms on various metals, such as Fe,^7–9^ Ni,^10–13^ Co,^14,15^ Cu(111),^16^ Pd(111),^17^ Pt,^18^ Rh(111),^19^ Ru(0001)^20–27^ and Ir.^28–32^ Boisen employed a model describing the catalytic trends over transition metal catalysts and found Ru to be the most active metal for this reaction.^33^ Egawa *et al.* investigated the desorption and kinetic process of NH_3_ decomposition on Ru surfaces using electron spectroscopy and diffraction techniques.^34^ They observed that the reaction takes place from 400 K reaching an equilibrium of H_2_ and NH_3_ in the gas phase at around 500 K, while the formation rate of N_2_ peaks at 570 K according to the thermal-desorption spectra. Mortensen *et al.* applied supersonic molecular beam techniques to study the dissociation of ammonia also on the Ru(0001) surface and proposed a mechanism dominated by the diffusion of intermediate species.^35^ The fcc structure of Ru also plays a crucial role in the hydrogen evolution reaction. Hanyu *et al.* described the compensation effect on 2 nm fcc Ru supported nanoparticles, where the turnover frequency (TOF) for ammonia borane hydrolysis reached 0.72 mol m^−2^ h^−1^ (more than 90% of the theoretical value) at 15 °C.^36^

Although Ru shows good activity for catalyzing this process, its scarcity makes its large scale implementation prohibitive unless it is used as dispersed fine nanoparticles. On the other hand, the price of Ir is relatively low, and it is currently employed to decompose similar molecules (*e.g.* N_2_H_4_) as propeller fuel in spaceships.^37^ George *et al.* reported that Ir catalysts have several orders of magnitude higher activity to decompose NH_3_ than other transition metals such as Pd, Pt and Rh at 750 K.^38^ Santra *et al.* carried out a temperature-programmed reaction study on Ir(100) and found that the associative nitrogen desorption is the crucial step for continuous and efficient ammonia decomposition.^29^ Huang *et al.* arrived at the same conclusion using computational methods on Ir(111) and Ir(110).^31^ They also suggested that the competition between desorption and dissociation can be tuned *via* the control of pressure and temperature during the reaction.

In order to develop more efficient catalysts, many experimental studies of ammonia decomposition on Ru and Ir catalysts focused on the relationship between the composition and atomicity of catalysts and product yields. Both temporal analysis of products (TAP)^39,40^ and steady-state isotopic transient kinetic analysis (SSITKA)^41–43^ can be applied to study the characteristics of the active sites and provide information on the adsorptions and reactions. García *et al.* carried out multi-pulse TAP experiments to understand the main mechanistic features involved in the catalytic decomposition of NH_3_ over carbon-supported Ru and Ir catalysts.^32^ The results suggested that the surface life-time of N species on the Ir surface is shorter than that on the Ru surface, leading to faster N_2_ desorption. John and co-workers found that NH_*x*_ species are the primary surface intermediates in the temperature range from 623 K to 673 K (204 kPa) and adsorbate N is the most abundant intermediate from 623 K to 773 K using SSITKA.^42^ To date, a systematic and detailed comparison of the exact mechanism and microkinetic model for NH_3_ decomposition on Ru and Ir supported nanoparticles is scarce in the literature, especially including the model describing the Ru fcc surface, which is observed in the Ru nanoparticle size range of 2.0–5.5 nm.^44^

Due to the complexity and difficulty in observing the adsorbed reaction intermediates, many aspects concerning the reaction processes at the atomic level remain unclear. For this reason, we have performed a density functional theory (DFT) investigation providing accurate information on all reaction species during ammonia decomposition on hcp Ru(0001), fcc Ru(111) and fcc Ir(111) and make a comparison with former data. We extended these results with microkinetic simulations, including batch reactor and temperature-programmed desorption simulations, hence, providing rates and selectivity information as a function of the catalysts' nature and closing the gap between modelling and experiments.

## 2. Computational details

### 2.1 DFT calculations

We employed the Vienna *Ab initio* Simulation Package (VASP) to simulate the NH_3_ decomposition reactions on Ru (hcp and fcc) and Ir metal catalysts.^45,46^ The spin-polarized revised Perdew–Burke–Ernzerhof (rPBE) method of the generalized gradient approximation (GGA) was adopted to describe the exchange–correlation with a plane-wave kinetic cutoff energy of 500 eV.^47^ Non-spherical contributions to atomic cores from the gradient corrections were represented by the projector augmented wave (PAW).^48–50^ The zero-damping DFT-D3 method was used to describe long-range interactions.^51^ The optimized convergence threshold of internal forces and electronic relaxation was set to 0.02 eV Å^−1^ and 10^−5^ eV, respectively. A 3 × 3 × 1 *k*-spacing Monkhorst–Pack grid sampled the Brillouin zone with a smearing broadening of 0.2 eV.

The optimized bulk lattice parameters are shown in Table 1. All surfaces were represented by a p(4 × 4) supercell slab model with five atomic layers, where the top three layers were fully relaxed and the bottom two were fixed at the optimized bulk lattice. We added 15 Å of vacuum perpendicular to the slab to avoid any spurious interaction with periodic images. Dipole correction perpendicular to the surface was applied upon the adsorption of any species. The molecular adsorption energies are defined using eqn (1), and the relative energies along the energy profiles are calculated using eqn (2).

1 *E*_ads_ = *E*_system_ − *E*_surface_ − *E*_molecule_

2
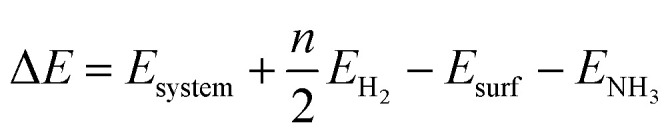
where *E*_system_ is the total energy of the adsorbed system, *E*_surface_ denotes the energy of the clean surfaces, and *E*_NH_3__ and *E*_H_2__ are the energy of the ammonia and the hydrogen isolated molecules. The half energy of a hydrogen molecule refers to the energy of one H atom, and *n* is the number of H dissociated from NH_3_.

**Table 1:** The bulk lattice parameters of Ru(hcp), Ru(fcc) and Ir(fcc)

Surface	This work	Previous studies	Experiments
Ru(hcp)	*a* = 2.691 Å, *c*/*a* = 1.572	*a* = 2.754 Å, *c*/*a* = 1.587 (ref. 52)	*a* = 2.706 Å, *c*/*a* = 1.582 (ref. 53)
Ru(fcc)	3.792 Å	3.825 Å (ref. 54), 3.830 Å (ref. 55)	—
Ir(fcc)	3.842 Å	3.876 Å (ref. 56)	3.839 Å (ref. 57)

The reaction energy (*E*_r_) is given by the energy difference of the final state (FS) and the initial state (IS) (eqn (3)). When the *E*_r_ value is negative, it means an exothermic step. The transition states (TS) were determined using the climb-image nudged elastic band (ci-NEB) combined with the improved dimer method (IDM) and ensuring a unique imaginary frequency along the reaction coordinate.^58–60^ We defined the forward and reverse activation barriers (*E*_a_) as the energy difference between the TS and IS and between the TS and FS, respectively (eqn (4) and (5)).

3 *E*_r_ = *E*^FS^ − *E*^IS^

4 *E*^forward^_a_ = *E*^TS^ − *E*^IS^

5 *E*^reverse^_a_ = *E*^TS^ − *E*^FS^

### 2.2 Microkinetic simulations

We constructed a kinetic model of the NH_3_ decomposition reaction based on a microcanonical ensemble within the transition state theory (TST) framework, which employs the Eyring and Evans and Polanyi approximation to compute the reaction constants of all surface elementary reactions (eqn (S24), (S26) and (S27) in the ESI†).^61–63^ Although the TST has weaknesses, it is widely used to provide useful information on the design of catalysts.^64,65^ Some of the TST weaknesses are that it assumes (i) no quantum tunnelling, (ii) the intermediates are long-lived to follow the Boltzmann distribution of energy, and (iii) all the species reaching the transition state evolve only to products. In the used model, the lateral adsorbate–adsorbate interactions are assumed to be negligible, *i.e.* low coverage, and mass transfer and diffusion do not limit the process kinetics. The partition functions to describe the thermodynamic properties as functions of temperature are listed in the ESI, eqn (S1)–(S12).† We have used numerical methods to solve the set of differential equations describing the relationship between the species, pressure and coverage, and time (listed in the ESI†).

## 3. Results and discussion

### 3.1 Surface species

**Fig. 1:**
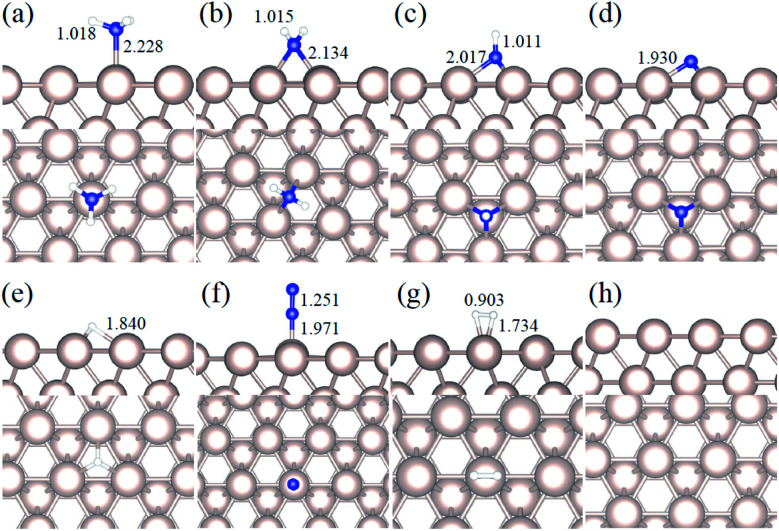
Side and top representation of the most favorable adsorption configurations on Ru(0001). (a) NH_3_, (b) NH_2_, (c) NH, (d) N, (e) H, (f) N_2_, (g) H_2_, (h) clean Ru(0001) surface. The insets show the average distances in Å. Blue, white and khaki balls refer to nitrogen, hydrogen and ruthenium atoms, respectively.

**Fig. 2:**
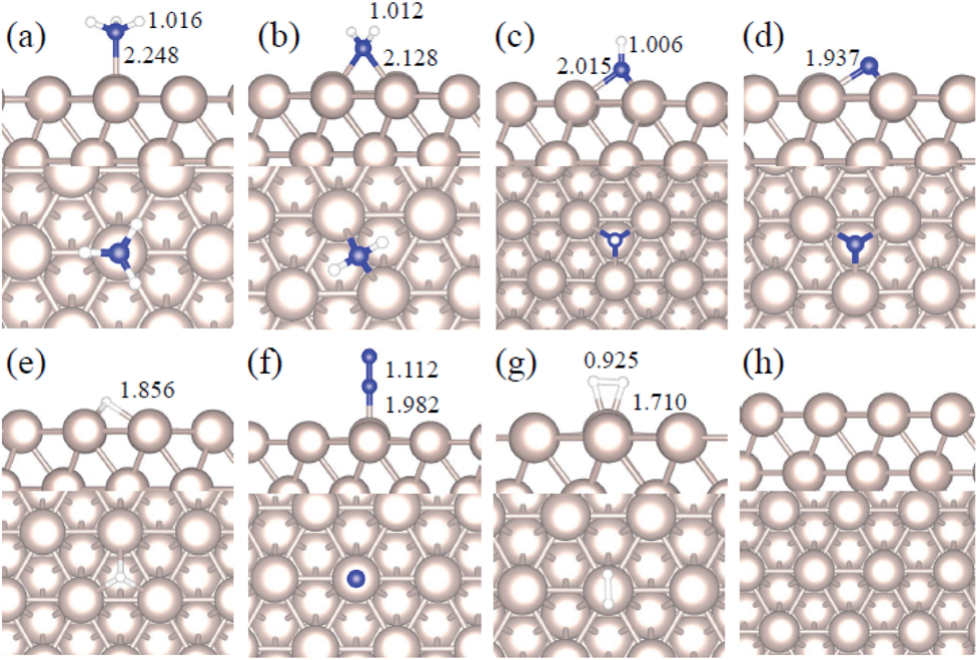
Side and top representation of the most favorable adsorption configurations on Ru(111). (a) NH_3_, (b) NH_2_, (c) NH, (d) N, (e) H, (f) N_2_, (g) H_2_, (h) clean Ru(111) surface. The insets show the average distances in Å. Blue, white and khaki balls refer to nitrogen, hydrogen and ruthenium atoms, respectively.

**Fig. 3:**
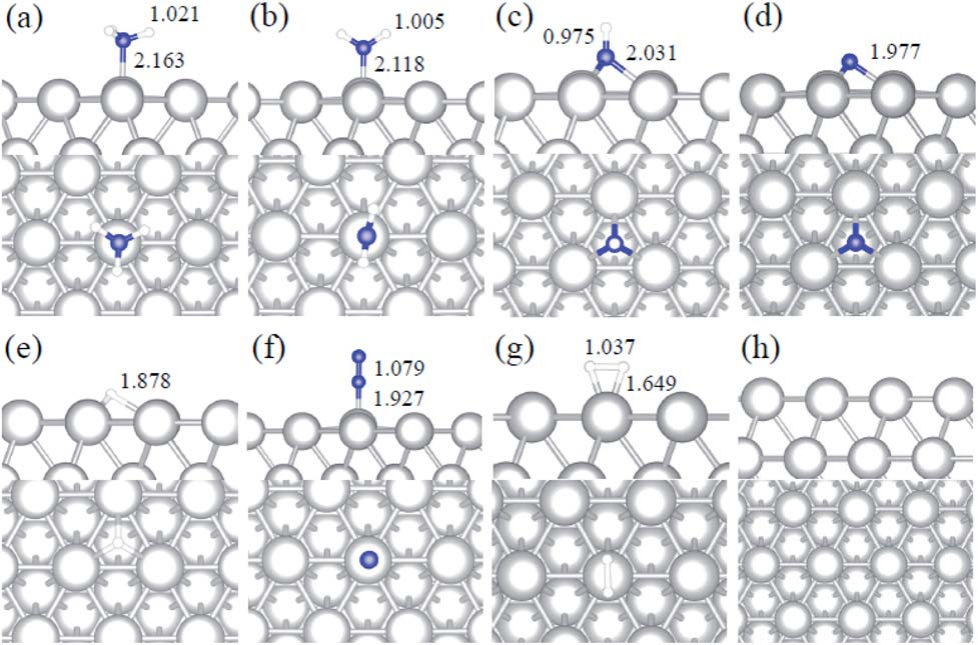
Side and top representation of the most favorable adsorption configurations on Ir(111). (a) NH_3_, (b) NH_2_, (c) NH, (d) N, (e) H, (f) N_2_, (g) H_2_, (h) clean Ir(111) surface. The insets show the average distances in Å. Blue, white and light grey balls refer to nitrogen, hydrogen and iridium atoms, respectively.

We studied all the non-equivalent adsorptions and configurations of surface species on Ru(0001), Ru(111) and Ir(111) in order to derive the reaction mechanism. Table 2 summarizes the most favorable adsorption properties of NH_*x*_ (*x* = 1–3), and atomic and molecular hydrogen and nitrogen. The adsorption modes are presented in Fig. 1, 2 and 3, respectively, for Ru(0001), Ru(111) and Ir(111). To investigate adsorbate's electrostatic structure, density of states (DOS) study and Bader charge analysis were carried out.^66^ The interaction of N lone pair of electrons with the d_*z*^2^_ orbital of the metals dominates the NH_3_ adsorption, with a charge transfer of 0.13 and 0.23 e^−^ from the Ru and Ir surface, respectively. The bond formation can also be observed at the projected density of states. The p_*z*_ and d_*z*^2^_ orbitals of N and the metals nicely overlap (ESI Fig. S11–S13†), over a broader energy range on Ir than on Ru. The capacity of Ir to provide more electron density to the bond with N than Ru is able to stabilize the NH_2_ intermediate on the top site while on Ru it falls to a bridge position. These adsorption trends are consistent with experimental findings using the scanning tunnelling microscopy method.^22^

**Table 2:** Adsorption energies (*E*_ads_) and average distances between the metal and nitrogen (*d*_TM–N_) and between nitrogen and hydrogens (*d*_N–H_) of NH_*x*_ (*x* = 1–3) and atomic and molecular H_2_ and N_2_ on (a) Ru(0001), (b) Ru(111) and (c) Ir(111) (T: top; B: bridge; hcp: hcp hollow; fcc: fcc hollow)

Species	Favorable site			*E*_ads_ (eV)			*E* ^zpe^_ads_ (eV)			*d*_N–H_ (Å)			*d*_TM–N_ (Å)		
	a	b	c	a	b	c	a	b	c	a	b	c	a	b	c
NH_3_	T	T	T	−0.98	−0.88	−1.19	−0.94	−0.84	−1.13	1.018	1.016	1.021	2.228	2.248	2.163
NH_2_	B	B	T	−0.48	−0.30	−0.32	−0.61	−0.44	−0.43	1.015	1.012	1.005	2.134	2.128	2.118
NH	hcp	hcp	fcc	−0.46	−0.24	0.09	−0.76	−0.53	−0.19	1.011	1.006	0.975	2.017	2.015	2.031
N	hcp	hcp	fcc	−0.85	−0.84	0.11	−0.83	−0.82	0.11	—	—	—	1.930	1.937	1.977
H	fcc	fcc	fcc	−0.56	−0.41	−0.33	−0.53	−0.39	−0.34	—	—	—	—	—	—
N_2_	T	T	T	−0.06	−0.55	−0.38	−0.01	−0.50	−0.31	—	—	—	1.971	1.982	1.927
H_2_	T	T	T	−0.40	−0.35	−0.36	−0.35	−0.28	−0.32	—	—	—	—	—	—

Generally, ammonia decomposition intermediates over Ru(0001) and Ru(111) present very similar behavior, except that the adsorption of N_2_ on Ru(111) is more favorable, attributed to the meta-stability of the fcc phase.

Ir(111) has the strongest NH_3_ adsorption compared with the Ru surfaces as it favors the electron back-donation with the adsorbed species. Along with the dehydrogenation of ammonia, the coordination of N with metal atoms increases, *i.e.* the adsorption site changes from top to bridge to hollow, and the perpendicular distance between N atoms and the surface decreases. These findings indicate that the interaction of N atoms with surfaces is strengthened with each dehydrogenation.

### 3.2 Reaction thermochemistry

**Fig. 4:**
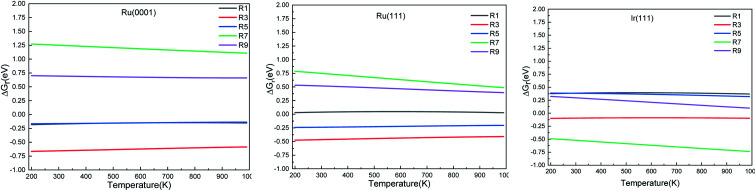
Free energy difference (Δ*G*_r_) of the elementary steps in ammonia dehydrogenation (R1–R5) and N_2_ and H_2_ formations (R7 and R9) as a function of temperature.

**Fig. 5:**
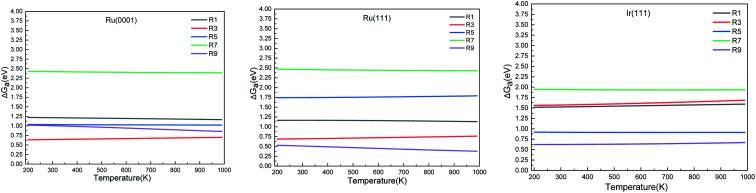
Activation energy (Δ*G*_a_) of the elementary steps in ammonia dehydrogenation (R1–R5) and N_2_ and H_2_ formations (R7 and R9) as a function of temperature.

We calculated the reaction energies (Δ*G*_r_) (Fig. 4) and the activation energies (Δ*G*_a_) as a function of temperature (Fig. 5) for each reaction step in the ammonia dehydrogenation (R1, R3 and R5 in Table 3) and in the N_2_ and H_2_ formations (R7 and R9 in Table 3). NH_3_ decomposition is thermodynamically favorable on Ru, but on Ir(111), it is limited by the dehydrogenation of NH_3_ (R1) and NH (R5). In particular, R1 presents a substantial activation energy, which aligns with the stability of the NH_3_ molecule over the surface. The most endothermic processes on Ru surfaces are the formations of N_2_ (R7) and H_2_ (R9). Indeed, the nitrogen recombination has been identified as the rate-determining step in both Ru(0001) and Ir(111).^67,68^ Interestingly, in Fig. 5, the activation energy for hydrogen evolution (R9) on Ru(111) is practically half of that on Ru(0001) and only slightly higher than on Ir(111). Such an energy difference explains the divergent results when comparing the H_2_ formation rates, *i.e.* Ru loading beyond a certain amount decreases the catalytic activity since it reduces the Ru fcc phase.^69^ Therefore, to improve the catalytic activity at low temperature, tuning the morphology of the catalyst is crucial.

**Table 3:** Elementary reactions and corresponding pre-exponential factors (*ν*) and reaction constants (*k*, in corresponding units) in the ammonia decomposition process over Ru(0001), Ru(111) and Ir(111) at 300 K

No.	Reaction	Ru(0001)		Ru(111)		Ir(111)	
		*ν*	*k*	*ν*	*k*	*ν*	*k*
A1	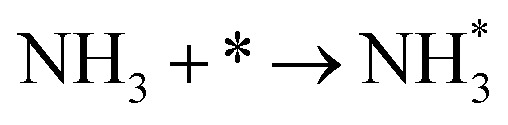	1.29 × 10^8^	48.09	1.28 × 10^8^	47.75	9.53 × 10^7^	35.60
D1	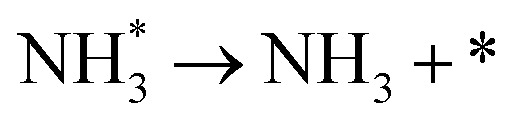	1.29 × 10^8^	3.53 × 10^−5^	1.28 × 10^8^	1.79 × 10^−3^	9.53 × 10^7^	3.60 × 10^−8^
R1	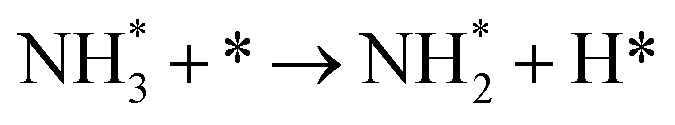	1.09 × 10^13^	4.2 × 10^−8^	5.37 × 10^12^	1.36 × 10^−7^	3.15 × 10^12^	7.63 × 10^−14^
R2	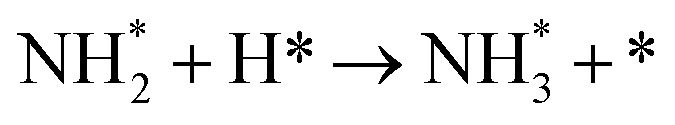	2.61 × 10^13^	1.38 × 10^−10^	1.12 × 10^13^	1.24 × 10^−6^	6.60 × 10^12^	4.73 × 10^−7^
R3	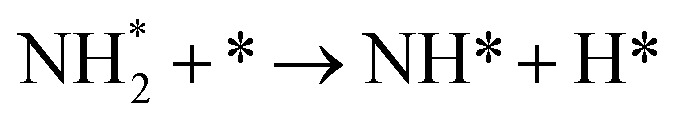	4.01 × 10^12^	60.34	3.95 × 10^12^	8.14	3.71 × 10^12^	1.48 × 10^−14^
R4	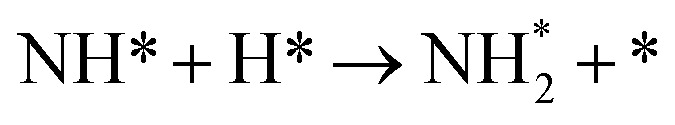	7.78 × 10^12^	1.15 × 10^−9^	7.51 × 10^12^	2.31 × 10^−7^	5.06 × 10^12^	5.26 × 10^−16^
R5	NH* + * → N* + H*	7.13 × 10^12^	2.97 × 10^−5^	6.01 × 10^12^	2.92 × 10^−17^	6.93 × 10^12^	2.86 × 10^−3^
R6	N* + H* → NH* + *	8.32 × 10^12^	6.17 × 10^−8^	7.19 × 10^12^	3.42 × 10^−21^	5.74 × 10^12^	6.68 × 10^3^
R7	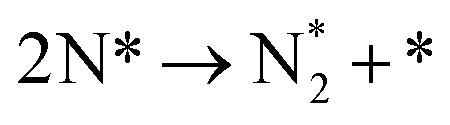	1.06 × 10^13^	2.11 × 10^−28^	1.08 × 10^13^	5.03 × 10^−29^	9.18 × 10^12^	2.13 × 10^−20^
R8	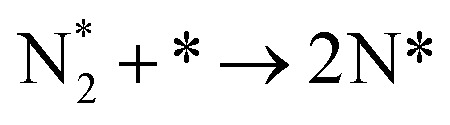	1.97 × 10^12^	4.08 × 10^−8^	5.48 × 10^11^	9.95 × 10^−18^	7.79 × 10^11^	3.24 × 10^−30^
D2	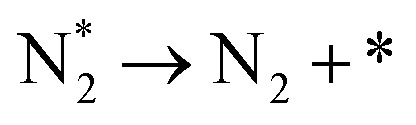	1.29 × 10^8^	1.24 × 10^12^	1.28 × 10^8^	410.62	9.52 × 10^7^	1.25 × 10^6^
A2	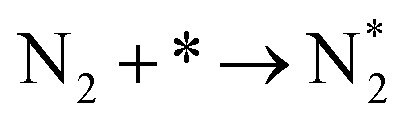	1.29 × 10^8^	1.26 × 10^4^	1.28 × 10^8^	1.25 × 10^4^	9.52 × 10^7^	9.34 × 10^3^
R9	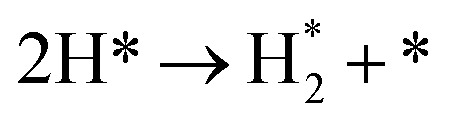	1.44 × 10^13^	1.86 × 10^−4^	1.79 × 10^13^	4.56 × 10^4^	6.25 × 10^12^	2.12 × 10^2^
R10	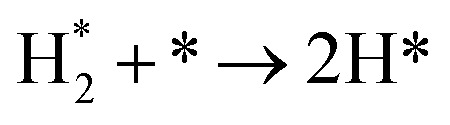	4.99 × 10^11^	6.21 × 10^10^	8.54 × 10^12^	1.12 × 10^13^	8.14 × 10^11^	2.65 × 10^6^
D3	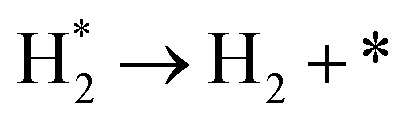	1.29 × 10^8^	7.04 × 10^3^	1.28 × 10^8^	4.84 × 10^4^	9.54 × 10^7^	3.86 × 10^2^
A3	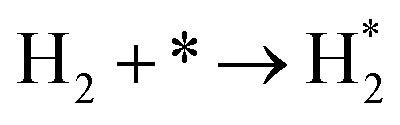	1.29 × 10^8^	7.29 × 10^2^	1.28 × 10^8^	7.23 × 10^2^	9.54 × 10^7^	5.39 × 10^2^

We have selected three different temperatures (*i.e.* 300, 600 and 900 K) and calculated the energetic profiles of the stoichiometric reaction (NH_3_ → 0.5N_2_ + 1.5H_2_), see Fig. S6–S8 in the ESI† where TS1, TS2 and TS3 are the transition states of the ammonia dehydrogenation process, and TS4 and TS5 are the transition states for the atomic recombination of nitrogen and hydrogen, respectively.

### 3.3 Reaction constants

We have derived the rate law's pre-exponential factors and reaction constants for every N–H dissociative step and adsorbate recombination based on the reaction energy profiles, see Table 3. Fully aligned with the discussion above is that the formation of adsorbate N_2_ has the smallest reaction constant indicating that it is the rate-determining step. Comparing the reaction constants for adsorbate N_2_ formation (R7) and its dissociation (R8), we can conclude that on Ru, the equilibrium is shifted towards the adsorbed atomic species; in contrast, it is shifted towards N_2_ on Ir. This result highlights the ability of Ir catalysts to promote N_2_ desorption.

### 3.4 Microkinetics

#### 3.4.1 Temperature-programmed desorption (TPD)

**Fig. 6:**
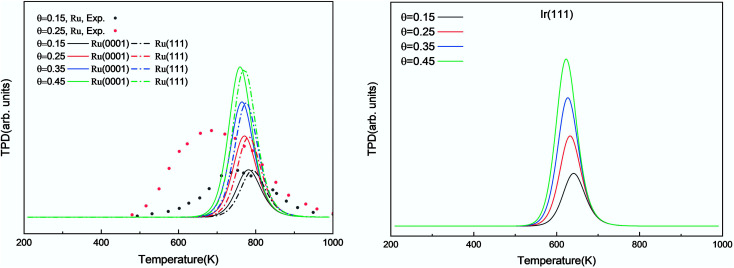
Simulated N_2_ TPD spectra on Ru(0001) and Ru(111) (left) and on Ir(111) (right) at different initial coverages (*θ* in ML) with a heating rate of 1 K min^−1^. The experimental data were extracted from ref. 70.

We investigated the individual desorption of N_2_ and H_2_ from the surfaces as a crucial step to complete the catalytic cycle. We found that the N_2_ TPD spectra (Fig. 6, left) on the two Ru surfaces are very similar. There is a ∼10 K shift to high temperature for nitrogen desorption on Ru(111) compared with Ru(0001). Compared with the experimental curve, the simulated TPD has a slight shift to higher temperatures.^70^ The reason for this deviation is that although we considered the N coverage effect to be negligible beyond 1/9 ML, it actually weakens the N adsorption considerably.^71^ This conclusion is derived from the agreement between the experiment and simulation at low coverage (*θ* = 0.15 ML). Another difference between the simulated and experimental N_2_ TPD is the width of the signal, which can be related to the lack of uniform nanoparticles and the temperature rate during the experiments.

**Fig. 7:**
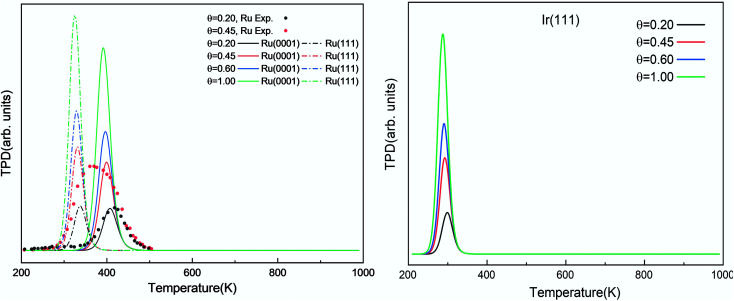
Simulated H_2_ TPD spectra on Ru(0001) and Ru(111) (left) and Ir(111) (right) at different initial coverages (*θ* in ML) with a heating rate of 1 K min^−1^. The experimental data were extracted from ref. 72.

The simulated H_2_ TPD patterns of Ru and Ir(111) surfaces are plotted in Fig. 7. The simulated data of Ru(0001) at 0.20 ML coverage fit the experimental research very well. However, at an H_2_ coverage of 0.45 ML, the experimental signal falls between the simulated patterns of hcp and fcc Ru surfaces, indicating the importance of nanoparticles' size and uniformity in the experiment. The match between simulation and experiments also implies a low effect of H coverage on the H adsorption energy. We can conclude that although the ammonia dehydrogenation on Ir is not as favorable as on Ru, the more favorable desorption of products makes it a suitable catalyst.

#### 3.4.2 Batch reactor simulation

**Fig. 8:**
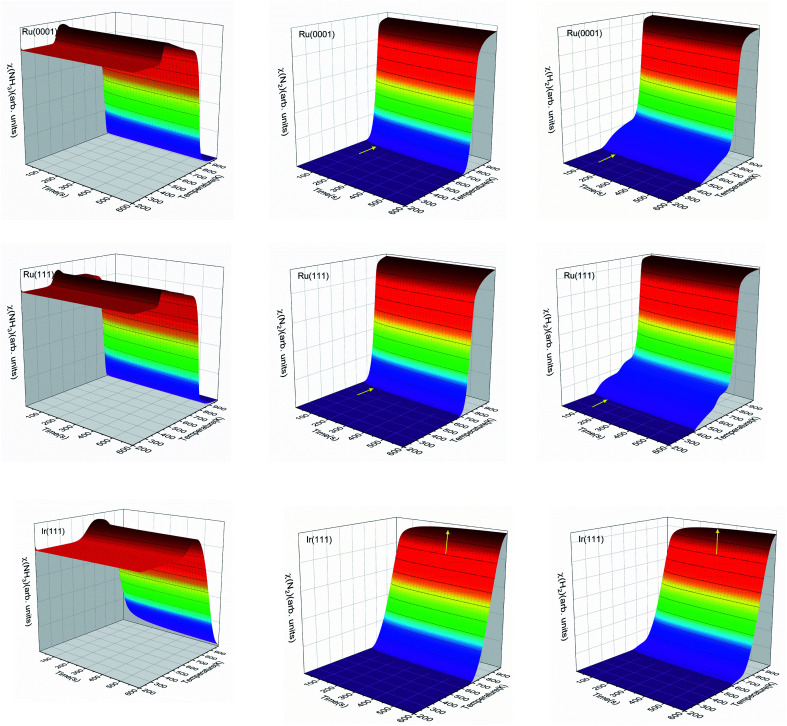
The ratios of molecular NH_3_, N_2_ and H_2_ with surface sites as functions of temperature and time on Ru(0001), Ru(111) and Ir(111) in batch reactor simulations. The initial ratio of NH_3_ : surface sites is 5 : 1. The inset yellow arrows indicate the stabilization of N_2_ and H_2_.

We have simulated the ratio between molecular species and active sites as a function of temperature and time, as shown in Fig. 8. At low temperatures, gas-phase NH_3_ will adsorb on the surface and saturate the free sites. Then, as the temperature increases, the adsorbed NH_3_ may react and desorb. The temperature range of the NH_3_ desorption process on Ru is from 400 to 450 K, while it is between 500 and 700 K on Ir(111). The NH_3_ desorption is observed in Fig. 8 with the increase of molecular NH_3_ before it decomposes. The NH_3_ contents on Ru(0001) and Ru(111) reach the steady-state in ∼100 s, but on Ir(111), this needs at least ∼300 s, which is seen in Fig. 8 for N_2_ and H_2_. The three surfaces generate molecular N_2_ at a temperature of ∼700 K. Ru(111) starts to produce H_2_ at ∼400 K, the lowest temperature among these three surfaces.

**Fig. 9:**
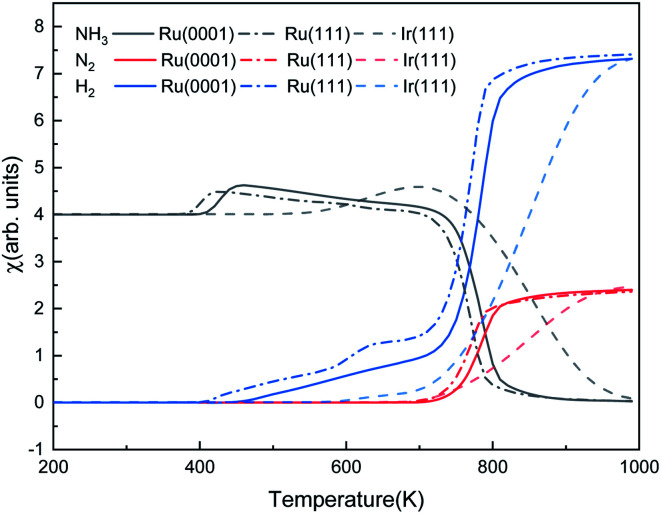
The steady-state ratios (*χ*) of NH_3_, N_2_, and H_2_ as functions of temperature on Ru(0001), Ru(111) and Ir(111) in batch reactor simulations. The initial simulated conditions are an NH_3_ ratio of 5 : 1 with a free surface. The reaction time is 600 s.

Fig. 9 shows the steady-state reaction details by depicting the NH_3_, N_2_, and H_2_ contents at 600 s as functions of temperature for the three surfaces. Ru(0001) has similar catalytic behavior to Ru(111): after a relatively slow evolution, the H_2_ production increases dramatically from 700 K and reaches a plateau at around 900 K. However, the H_2_ production on Ru(111) takes place at 400 K, while on Ru(0001), it is at 425 K. Our simulation results suggest that ammonia dissociates on Ir(111) at above 500 K. These results are consistent with the low- and high-temperature profiles for the decomposition of hydrazine (N_2_H_4_) on Ir(111), *i.e.* at temperatures below 500 K, the products of hydrazine decomposition are mainly NH_3_ and N_2_; however, NH_3_, N_2_ and H_2_ are observed above 500 K.^73,74^

**Fig. 10:**
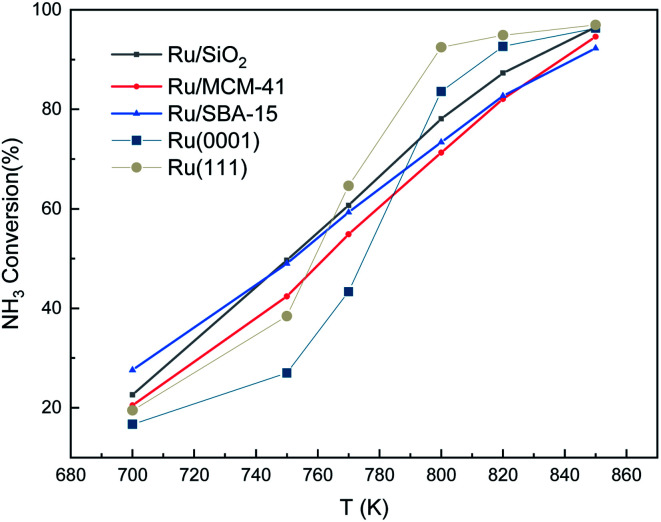
Ammonia conversion (in %) over supported Ru catalysts. Experimental trends were obtained from ref. 69. The initial simulated conditions are an NH_3_ : surface ratio of 5 : 1. The reaction time is 600 s.

We also made a comparison between experimental and simulated ammonia conversion on Ru catalysts, Fig. 10.^69^ Below 770 K, the results of Ru(111) fit the experimental data well, since above that temperature, the existing fcc Ru moieties may reconstruct to hcp and sinter to larger structures.^75^ Notice that Ru hcp fits better at high temperature. However, the NH_3_ conversion in the simulated process increases faster than the experimental one. This discrepancy between simulations and experiments may be due to the coverage effect of N, *i.e.* the NH_3_ decomposition reaction becomes more favorable at high N coverages as discussed in the TPD section.

**Fig. 11:**
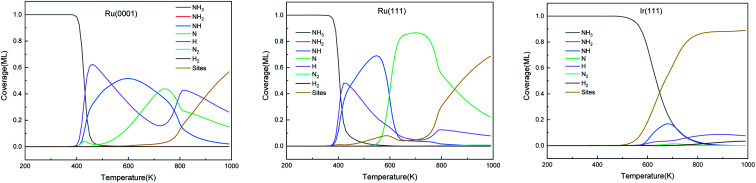
The steady-state of surface species distribution on Ru(0001), Ru(111) and Ir(111) surfaces in the temperature range of 200–1000 K at 600 s.

The predominant species on the surfaces at the steady-state (time is 600 s) within a temperature range of 200–1000 K are plotted in Fig. 11. Ru surfaces have a wider variety of surface species than Ir(111). As shown in Fig. 11, NH_3_, NH, N and H are the main predominant species with high coverages on Ru surfaces during the NH_3_ decomposition process. On Ru(0001), H is the dominant species at 430–535 K, and at 700 K, atomic N accumulates on the surface and replaces NH as the main surface species. The notable species on Ru(111) during the reaction are NH and N at 445–600 K and 600–850 K, respectively. Since Ru(111) has a lower Δ*G*_r_ for H_2_ evolution (R9) than Ru(0001), H is the predominant species on Ru(111) at a narrow temperature range of 410–445 K. In contrast, the Ir(111) surface presents considerable contents (>0.1 ML) of only NH_3_ and NH as the dissociation of NH_3_ starts at 500 K; NH is the predominant species on the surface in the temperature range of 550–760 K with a maximum coverage of 0.17 ML at 680 K. Owing to the low Δ*G*_a_ of R7, atomic N does not accumulate on the Ir(111) surface.

## 4. Conclusions

We carried out a mechanistic investigation of NH_3_ decomposition on hcp Ru, fcc Ru and fcc Ir using DFT-D3. The most favorable adsorption sites range from NH_3_ being on top, to bridge, and to hollow sites, with every dehydrogenation strengthening the N bonding to the surface. The energy profiles show that the rate-limiting step is the atomic nitrogen recombination on all the surfaces studied, and although the NH_3_ dehydrogenation on Ir(111) is not as favorable as on Ru, the N_2_ desorption indicates that it is a promising catalyst candidate. We derived the free energies of each gas-phase and surface species between 200 and 1000 K by including entropic and specific heat contributions to the DFT energy. We implemented these free energies in a microkinetic model where the TPD experiment showed that both Ru surfaces, *i.e.* (0001) and (111), have similar desorption properties. The simulated TPD also proved to be useful in assessing the importance of N coverage on the model, *i.e.* the desorption shifts to lower temperatures with increasing N coverage. Batch reaction simulations described the reaction processes with the increase in temperature and time and indicated that Ru(111) produces H_2_ at a lower temperature than Ru(0001). On the Ir(111) surface, the dehydrogenation starts at higher temperatures than on Ru, but the desorption of N_2_ takes place at a lower temperature. The comparison between these results and experiments demonstrates that microkinetic simulations based on DFT results are a useful tool to investigate heterogeneous catalytic reactions and design novel catalysts.

## Conflicts of interest

There are no conflicts to declare.

## Supplementary Material

NA-003-D1NA00015B-s001
