# Supplementary material for: Kinetic and mechanistic analysis of NH3 decomposition on Ru(0001), Ru(111) and Ir(111) surfaces
Source: Nanoscale Adv. 2021 Feb 9;3(6):1624–32. doi: 10.1039/d1na00015b (PMC9418880; doi:10.1039/d1na00015b)
Supplement: NA-003-D1NA00015B-s001 [file NA-003-D1NA00015B-s001.pdf]

Electronic Supplementary Material (ESI) for Nanoscale Advances.
This journal is © The Royal Society of Chemistry 2021

Supporting Information

Kinetic and Mechanistic Analysis of NH_3 Decomposition on Ru(0001), Ru(111) and Ir(111) Surfaces

Xiuyuan Lu[a], Jing Zhang[a,b], Wen-Kai Chen[b], Alberto Roldan[a]

[a] Cardiff Catalysis Institute, School of Chemistry, Cardi□ University, Main Building, Park Place, Cardi□, CF10 3AT, UK

[b] College of Chemistry, Fuzhou University, Fuzhou, Fujian 350116, China

Ⅰ. Thermodynamic calculation

Calculation of thermodynamic properties (entropy, enthalpy and free energy) under a series temperature is the first step to construct a kinetic model. We programmed python scripts to achieve the calculation of partition functions and thermodynamic properties, as the flow diagram *Figure S1* below, furthermore, the properties at high temperature were extrapolated from the optimized data at 0K.

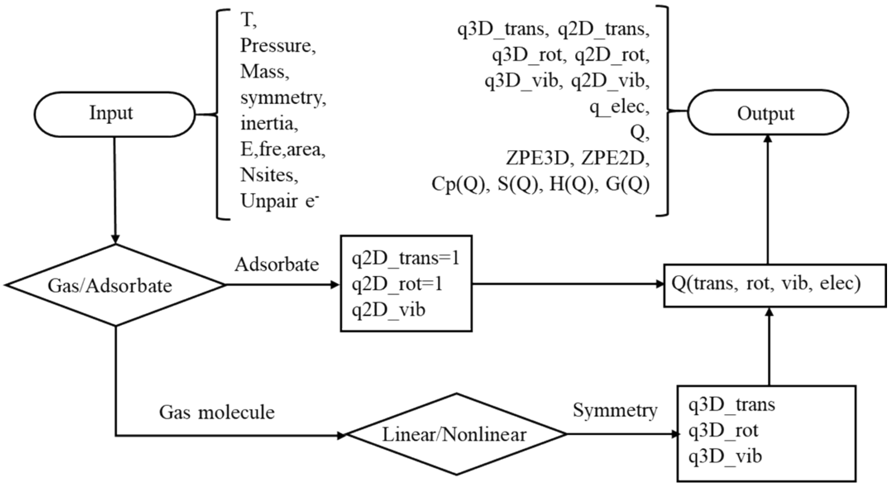

Figure S1. The flow diagram of thermodynamic calculation

To improve the accuracy of the energy, the zero-point energies (ZPE) are used to correct the static DFT electronic energy. ZPE refers to the vibrational energies that exist even at 0 K and is calculated as *Eq S1* where v_i accounts for the vibrational modes of the species.

$$ZPE = \sum_{i}^{n} \frac{1}{2} h v_i \qquad Eq\ S1$$

The temperature effect on adsorption energy (E_{ads}), reaction energy (E_r), activation energy (E_a) is taken into account to obtain more accurate results. The global

partition function, Q, is used to depict the energy as a function of temperature for the intermediates on the surface or in the gas phase, and the basic thermodynamic characters such as entropy (S), specific heat at constant pressure (C_p) and enthalpy (H) can be derived by Q as the following equations:

$$S = klnQ + kT\left(\frac{\partial lnQ}{\partial T}\right)_V \quad Eq\ S2$$

$$C_p = T\left(\frac{\partial S}{\partial T}\right)_P \quad Eq\ S3$$

$$H = E_{DFT} + E_{(S=0,T=0,ZPE)} + \int_0^T C_p \partial T \quad Eq\ S4$$

$$G = H - TS \quad Eq\ S5$$

Where k is Boltzmann constant, T is the temperature. The global partition function is calculated as *Eq S6*.

$$Q = q_{translational} * q_{rotational} * q_{vibrational} * q_{electronic} * q_{nuclear} \quad Eq\ S6$$

Translational, rotational, vibrational, electronic and nuclear contributions are considered. Normally, the electronic systems are in a single electronic state, and the nuclear partition functions are unity, i.e. $q_{electronic}$, $q_{nuclear}$ equal constant 1. The vibrational partition function of a system is obtained via *Eq S7*,

$$q_{vib} = \prod_{i=1}^{N} \frac{1}{1 - e^{-hv_i/k_BT}} \quad Eq\ S7$$

Where i is a specific vibrational mode and N is the number of vibrations. The vibrational partition function in the gas phase, q^{gas}_{vib}, is also calculated using the equation above for 3N_i-6 and 3N_i-5 vibrational degrees of freedom of a non-linear and linear molecule in the gas phase, respectively, where N_i is the number of atoms in the molecule. The 2D-translational partition function for a free molecule is derived by the Eq S8.

$$q^{2D}_{translation}(A,T) = \left(\frac{2\pi mk_BT}{h^2}\right)A_{cat} \quad Eq\ S8$$

Where A_{cat} is the average area of one active site on a catalyst.

The 3D-translational partition function for a molecule is calculated by the Eq S9, where $V_{(P,T)}$ is derived by $V_{(P,T)} = k_BT/p$, p is the pressure of the gas phase.

$$q^{3D}_{trans} = V_{(P,T)} * \frac{(2\pi mk_BT)^{3/2}}{h^3} \quad Eq\ S9$$

The Rotational partition function for a free molecule is calculated using Eq S10 and Eq S11, depending on its symmetry and linear type.

$$q^{linear}_{rot} = \frac{\pi^{1/2}}{\sigma h}(I_{ZZ})^{1/2}\left(8\pi^2k_BT\right)^{1/2} \quad Eq\ S10$$

$$q_{rot}^{nonlinear} = \frac{1}{\sigma}\left(\frac{8\pi^2 k_B T}{h^2}\right)^{3/2}\sqrt{\pi I_a I_b I_c} \qquad Eq\ S11$$

Where σ is the symmetry factor and I is the moment of inertia defined as *Eq S12*,

$$I = \sum_i m_i r_i^2 \qquad Eq\ S12$$

Where the sum is over the atoms in the molecule, m_i is the mass of atom and r_i is its distance from the rotation axis.

The thermodynamic properties in our system were calculated according to the above equations.[1] To evaluate the accuracy of our method, a comparison between calculated and standard thermodynamic properties of NH_3, N_2, H_2 was carried out. The reference properties are from the NIST database and Thermochemical Data of Pure Substances.[2,3]

Table S1 contains the data we used in the thermodynamic calculations, including the gas pressure, the electronic energy, the moment of inertia and vibration frequency from VASP calculation and the mass and symmetry of molecules.

Table S1 Optimized gas molecule information

Species	Energy(eV)	Vibration(cm^{-1})	Mass(kg)	Pressure(Pa)	Symmetry	Inertia($kg*m^2$)
H_2	-7.047	4360.85	3.35E-27	101325	2	2.35E-48
N_2	-17.0944	2006.95	4.65E-26	101325	2	7.82E-47
NH_3	-20.0541	3504.75,3503.83, 3372.67,1633.87, 1633.20,1035.29	2.83E-26	101325	3	3.51E-140

Figure S2, Figure S3 and Figure S4 show both the calculated and reference thermodynamic data between 300-1000K. According to the Gibbs energy calculated, the delta Gibbs energy of ammonia synthesis reaction was given. The delta Gibbs energy of calculation is corrected by experimental formation enthalpy of ammonia and pure gas, which is -45.94 kJ/mol and 0 kJ/mol. Since the absolute values of the delta Gibbs energy are small at 400 and 500K, the errors turned a little bit. Generally, our method based on VASP optimized results to calculate thermodynamic properties was proved accurately.

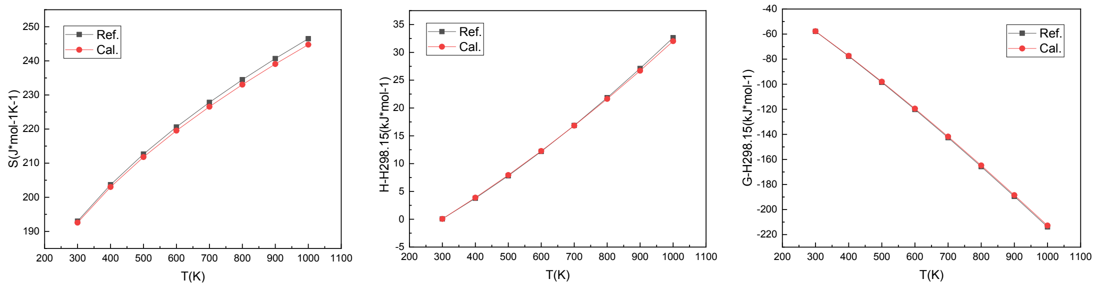

Figure S2. Calculated thermodynamic properties of gas phase NH3 compared with reference data from 300-1000K

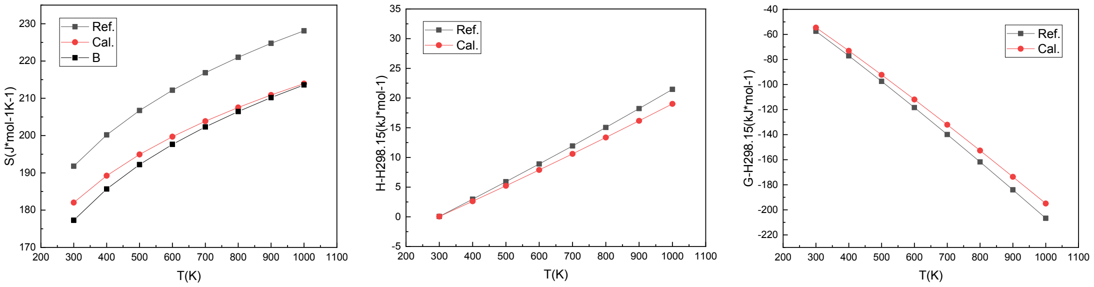

Figure S3. Calculated thermodynamic properties of gas phase N_2 compared with reference data from 300-1000K

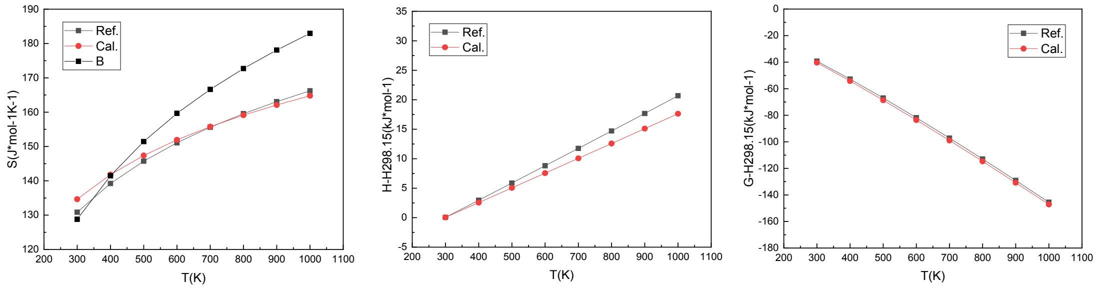

Figure S4. Calculated thermodynamic properties of gas phase H_2 compared with reference data from 300-1000K

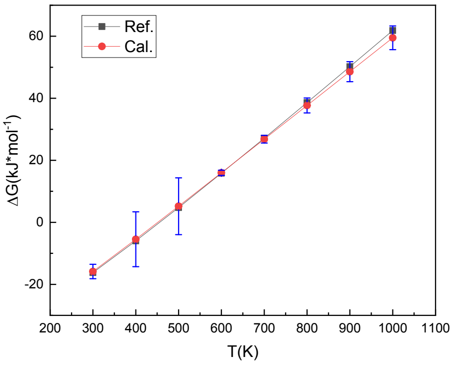

Figure S5. The delta Gibbs energy of ammonia synthesis reaction with error bar. The error was calculated by $(G_{cal} - G_{reference})/G_{reference} * 100$.

Ⅱ. Reaction thermochemistry

The recombination of dissociated N to nitrogen molecule on the surface (R7) needs to overcome a barrier (E_{a4}) of 2.40-2.62 eV at the temperature explored, which is supposed to be the rate-determining step. R5 and R9 carry out the NH dehydrogenation and the H re-coupling have close barrier energy at 300K, which are 1.03 eV and 1.01 eV respectively. Furthermore, only the N and H atoms recombination (R7 and R9) are endothermic elementary steps needing energy of 1.25 eV and 0.69 eV under 300K. With the rise of temperature from 300K to 900K, the dehydrogenation energy barrier (E_{a1}) of R1 decrease from 1.22 eV to 1.17 eV, indicating that high temperature is favourable to the first step dehydrogenation. Conversely, the energy barrier (E_{a2}) of R3 up from 0.64 eV to 0.69 eV with the rise of temperature. As the energy barrier to form NH by NH_2 (R3) dehydrogenation is relatively small. The lifetime of NH_2 is very short and detect it experimentally will be extremely challenging on Ru(0001).

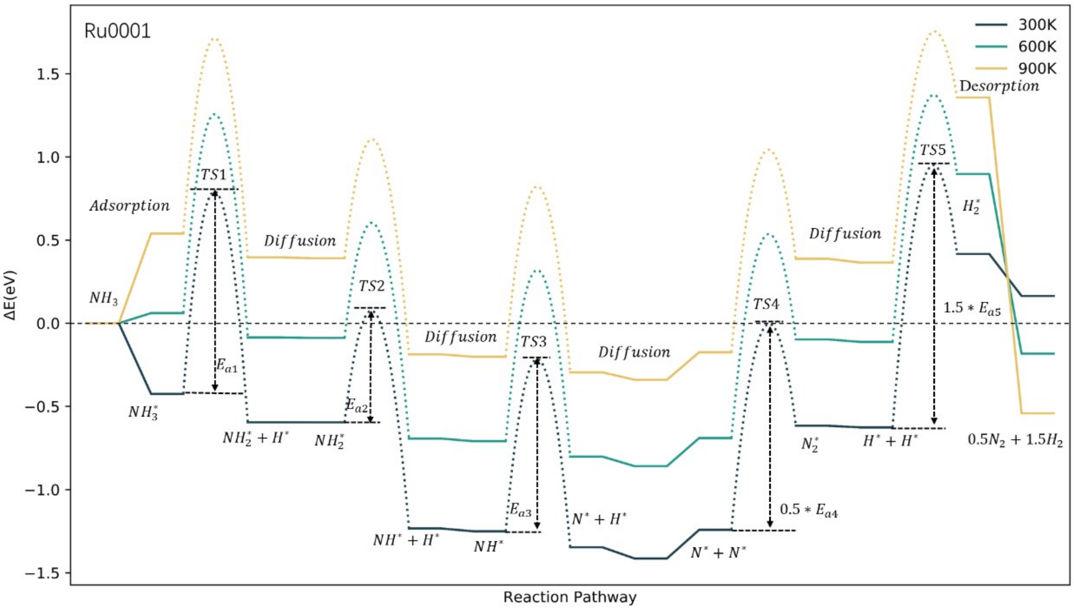

Figure S6. Energy profile of ammonia decomposition on Ru(0001) under 300K, 600K and 900K

Figure S7 depicts the energy profile of ammonia decomposition on Ru(111) at 300K, 600K and 900K. It suggests that the N_2 formation, R7, is the rate-determining step under the studied temperature proceeding barrier energy (E_{a4}) of 2.46 to 2.43 eV. The third dehydrogenation step (R5) to form N and H atoms, has the second-highest barrier energy (E_{a3} = 1.74eV) at 300K, and with the temperature increasing to 900K, it rises to 1.78 eV. The dehydrogenation of NH_3 has the third-highest barrier energy under 300 K which is 1.17 eV and decrease with the temperature. Comparing with Ru(0001), R5 on Ru(111) has higher barrier energy for generating N^* and H^*, but

the H_2 molecule generation (R9) becomes easier by overcoming barrier energy of 0.51 eV to 0.39 eV under 300 to 900 K. The shrinkage of barrier energy suggests that high temperature is favourable for the hydrogen generation thermodynamically. E_{a2}(0.70 eV) for R3 is close with E_{a5} for R9 under 300 K, while it will increase to 0.75 eV when temperature up to 900K.

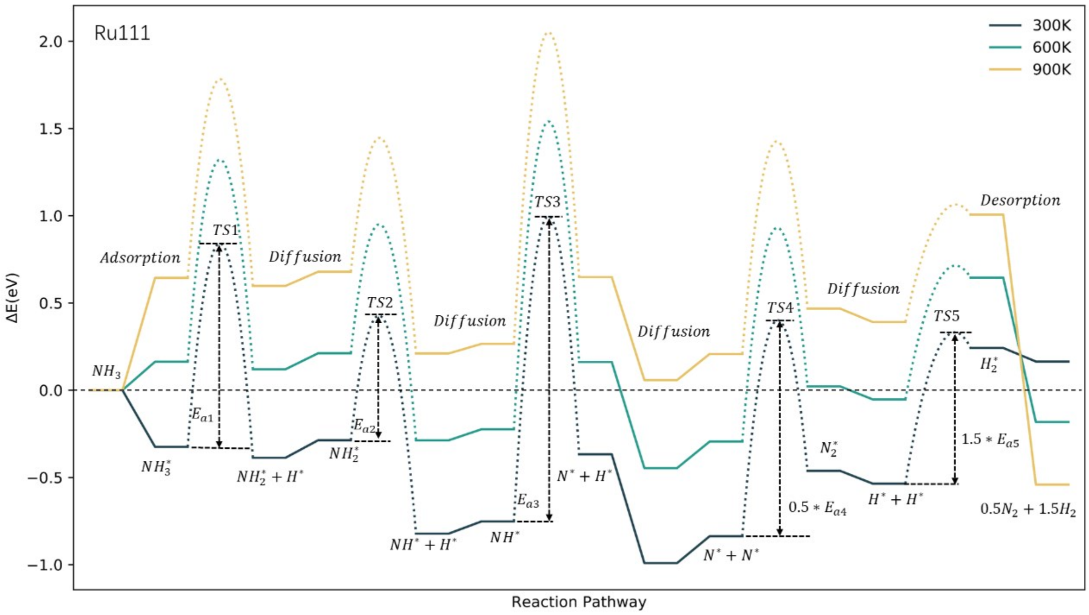

Figure S7. Energy profile of ammonia decomposition on Ru(111) under 300K, 600K and 900K

Figure S8 shows the energy profile of ammonia decomposition on Ir(111) at 300K, 600K and 900K. It suggests that nitrogen recombination, R7, is the rate-determining step as well, with barrier energy (E_{a4}) of 1.94 eV at studied temperature, which is close to previous research (1.75 eV for Ir(110)).[29] The first (R1) and second (R3) dehydrogenation of NH_3 needs to overcome similar barriers of around 1.55 eV at 300 K, which are increasing with the temperature. The last dehydrogenation step, R5, yields N^* and H^* by surpassing a 0.91 eV barrier at 300-900K, which is a moderate elementary step. The recombination of H^*, R9, is impaired by the rise of temperature since the barrier energy (E_{a5}) rise from 0.62 eV at 300 K to 0.66 eV at 900K.

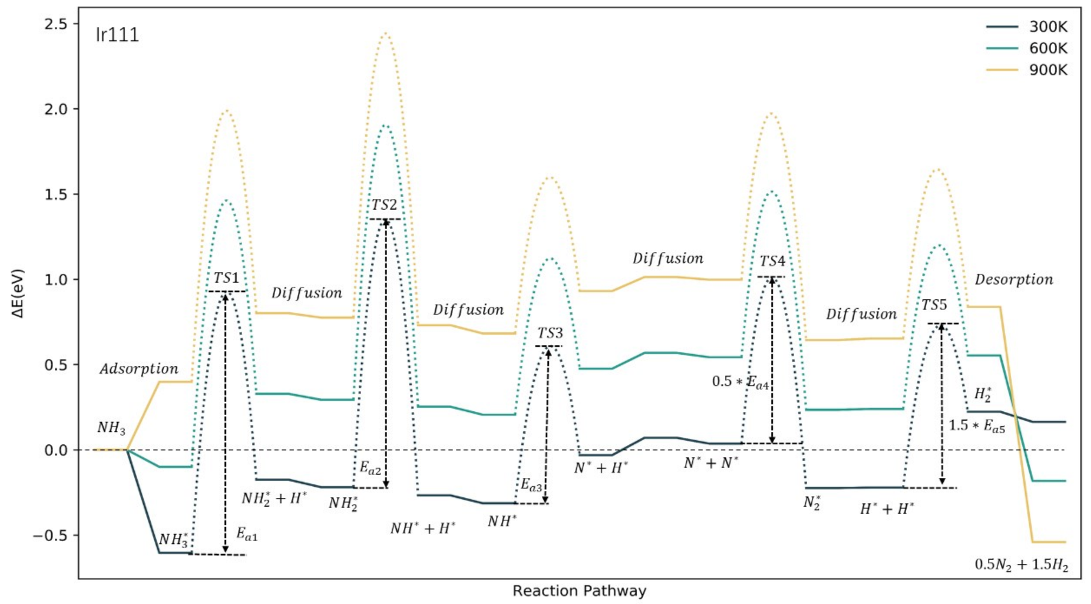

Figure S8. Energy profile of ammonia decomposition on Ir(111) under 300K, 600K and 900K

Ⅲ. Equilibrium constant and Reaction rate constant calculations

A script reading the partition functions, thermodynamic properties was implemented to calculate the equilibrium constant and reaction rate constant of all elementary steps. *Figure S9* shows the process of the script.

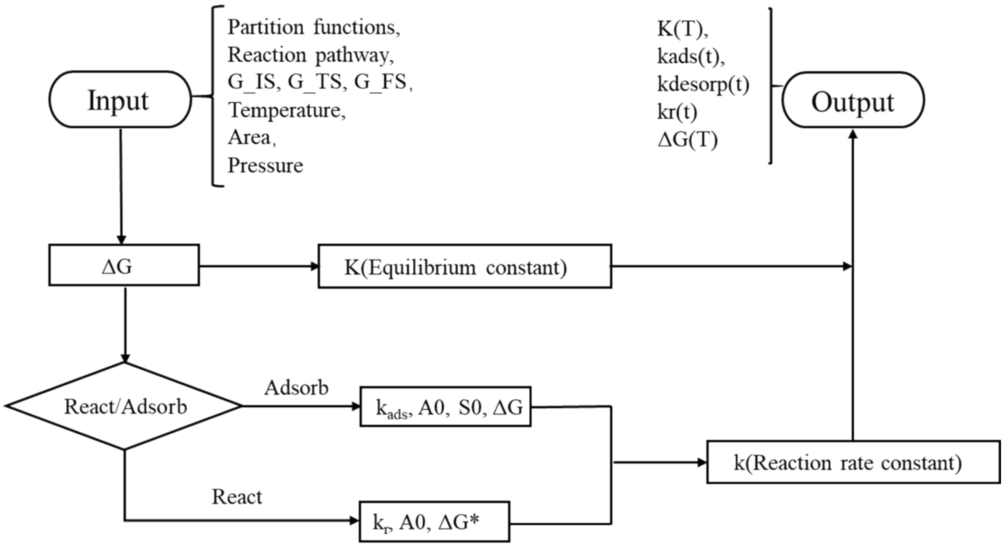

Figure S9. The flow diagram of reaction rate constant and equilibrium constant calculation

The barrier energy of adsorption and desorption processes are calculated by Eq S 13, Eq S 14 and Eq S 15:

$$E_a^{ads} = E_{ts} - E_{gas} - E_{surf} \qquad \text{Eq S 13}$$

$$E_a^{des} = E_{ts} - E_{ads} \qquad \text{Eq S 14}$$

$$E_{ts} = E_{gas} + E_{surf} + \left(ZPE_{2D}^{gas} - ZPE_{3D}^{gas}\right) \qquad \text{Eq S 15}$$

The adsorption energy is calculated by Eq S 16:

$$E_r = E_{gas} + E_{surf} - E_{ads} \qquad \text{Eq S 16}$$

The classical Hertz-Knudsen equation was employed to estimate the rate of adsorption, as following Eq S 17 to Eq S 18.

$$Q_{reactants} = \left(q_{trans2D}^{ads} * Q_{notrans3D}^{gas}\right)^{(stoichio_{ads})} * Q_{surf}^{(stoichio_{surf})} \qquad \text{Eq S 17}$$

$$Q_{ts} = \left(q_{vib2D}^{ads}\right)^{(stoichio_{ads})} * Q_{surf}^{(stoichio_{surf})} \qquad \text{Eq S 18}$$

$$Q_{reactants} = \left(Q_{3D}^{ads}\right)^{stoichio_{ads}} \qquad \text{Eq S 19}$$

$$Sticky_{ads} = \frac{Q_{ts}}{Q_{reactants}} * e^{-\frac{E_a^{ads}}{k_B T}} \qquad \text{Eq S 20}$$

$$Sticky_{des} = \frac{Q_{ts}}{Q_{products}} * e^{-\frac{E_a^{ads}}{k_B T}} \qquad \text{Eq S 21}$$

$$A_0 = areaS * \frac{1}{\left(2 * \pi * Mass * k_B * T\right)^{0.5}} \qquad \text{Eq S 22}$$

$$k_{ads/des} = A_0 * Sticky_{ads/des} \qquad \text{Eq S 23}$$

Where A_0 is the pre-exponential factor. The sticking coefficient, Sticky, is a measure of the fraction of incident molecules which adsorb upon the surface and is calculated via Eq S 20 and Eq S 21.

As for the ***surface reactions*** in the heterogeneous catalytic system, which is considered in our research, the constant rate (k) of each surface elementary step is commonly computed using the transition state theory (TST) approximation of Eyring and Evans and Polanyi, as follows:

$$k_r = A_0 e^{-\frac{\Delta G^*}{k_B T}} = \frac{k_B T q_{TS}}{h \ q_{IS}} e^{-\frac{\Delta G^*}{k_B T}} \quad \text{Eq S24}$$

Where h is the Plank constant, kB is the Boltzmann constant, T is the temperature, A_0 is the pre-exponential factor, ΔG* is the reaction activation energy, and q_{TS} and q_{IS} are the partition functions of reactants and transition states respectively. The translations and rotations of the adsorbed species are frustrated on the surface and therefore we considered only vibrational modes.

We have considered an active site as a hexagonal site where the reactants and products in every elementary step occupy only one site on the surface. Consequently, the coverage of free sites, $\theta_*(t)$, is defined by:

$$\theta_*(t) = 1 - \sum_i^n \theta_i(t) \quad \text{Eq S25}$$

Where the $\theta_i(t)$ represents the coverage of the intermediates present in the reaction system.

IV. The reaction and rate equations

The adsorption and first dehydrogenation of NH_3 are highly exothermic and the desorbed hydrogen and nitrogen molecules are assumed to leave from the surface immediately, therefore, the dissociated adsorption model is applied in the adsorption and desorption process of molecules. All the elementary steps in the ammonia decomposition and their rate equations are listed below.

Table S2. Elementary steps in the ammonia decomposition

No	Reaction	Rate equation
A1	$NH_3 + * \longrightarrow NH_3^*$	$r_{a1} = k_{a1} Y_{NH_3}(t) \theta_*(t)$
D1	$NH_3^* \longrightarrow NH_3 + *$	$r_{d1} = k_{d1} \theta_{NH_3}(t)$
R1	$NH_3^* + * \longrightarrow NH_2^* + H^*$	$r_{r1} = k_{r1} \theta_{NH_3}(t) \theta_*(t)$
R2	$NH_2^* + H^* \longrightarrow NH_3^* + *$	$r_{r2} = k_{r2} \theta_{NH_2}(t) \theta_H(t)$
R3	$NH_2^* + * \longrightarrow NH^* + H^*$	$r_{r3} = k_{r3} \theta_{NH_2}(t) \theta_*(t)$
R4	$NH^* + H^* \longrightarrow NH_2^* + *$	$r_{r4} = k_{r4} \theta_{NH}(t) \theta_H(t)$
R5	$NH^* + * \longrightarrow N^* + H^*$	$r_{r5} = k_{r5} \theta_{NH}(t) \theta_*(t)$
R6	$N^* + H^* \longrightarrow NH^* + *$	$r_{r6} = k_{r6} \theta_N(t) \theta_H(t)$

R7	$2N^* \rightarrow N_2^* + *$	$r_{r7} = k_{r7}\theta_N^2(t)$
R8	$N_2^* + * \rightarrow 2N^*$	$r_{r8} = k_{r8}\theta_{N_2}(t)\theta_*(t)$
D2	$N_2^* \rightarrow N_2 + *$	$r_{d2} = k_{d2}\theta_{N_2}(t)$
A2	$N_2 + * \rightarrow N_2^*$	$r_{a2} = k_{a2}Y_{N_2}(t)\theta_*(t)$
R9	$2H^* \rightarrow H_2^* + *$	$r_{r9} = k_{r9}\theta_H^2(t)$
R10	$H_2^* + * \rightarrow 2H^*$	$r_{r10} = k_{r10}\theta_{H_2}(t)\theta_*(t)$
D3	$H_2^* \rightarrow H_2 + *$	$r_{d3} = k_{d3}\theta_{H_2}(t)$
A3	$H_2 + * \rightarrow H_2^*$	$r_{a3} = k_{a3}Y_{H_2}(t)\theta_*(t)$

Where $Y_{molecule}(t)$ is the time-dependent ratio of the molecule and free sites.

Ⅳ. Differential equations in the TPD simulation of hydrogen and nitrogen

Temperature programmed reaction model start from pre-adsorbed NH_3, the temperature increase at a different rate from 200 to 1000 K while any gas was extracted to avoid the re-adsorption of gases. It is applied to examine the adsorption properties of N_2 and H_2 over different surfaces in our research.

$$\frac{dY_{N_2}}{dt} = r_{d2}$$

$$\frac{dY_{H_2}}{dt} = r_{d3}$$

$$\frac{d\theta_N}{dt} = r_{r5} - r_{r6} - 2 * r_{r7} + 2 * r_{r8}$$

$$\frac{d\theta_H}{dt} = r_{r1} - r_{r2} + r_{r3} - r_{r4} + r_{r5} - r_{r6} - 2 * r_9 - 2 * r_{10}$$

$$\frac{d\theta_*}{dt} = -\left(\frac{d\theta_N}{dt} + \frac{d\theta_H}{dt}\right)$$

The initial parameter is input into the program by the command:

YN_2, YH_2, θ_N, θ_H, θ_* = [0, 0, a, b, 1]

V. Differential equations in the reactor simulation

A batch reactor model under a variety of conditions is employed to investigate the catalytic properties when the metallic surface is in contact with a given pressure of NH_3.

$$\frac{dY_{NH_3}}{dt} = - r_{a1} + r_{d2}$$

$$\frac{dY_{N_2}}{dt} = - r_{a2} + r_{d2}$$

$$\frac{dY_{H_2}}{dt} = - r_{a3} + r_{d3}$$

$$\frac{d\theta_{NH_3}}{dt} = r_{a1} - r_{d1} - r_{r1} + r_{r2}$$

$$\frac{d\theta_{NH_2}}{dt} = r_{r1} - r_{r2} - r_{r3} + r_{r4}$$

$$\frac{d\theta_{NH}}{dt} = r_{r3} - R_{r4} - R_{r5} + R_{r6}$$

$$\frac{d\theta_{N}}{dt} = r_{r5} - r_{r6} - 2 * r_{r7} + 2 * r_{r8}$$

$$\frac{d\theta_{H}}{dt} = r_{r1} - r_{r2} + r_{r3} - r_{r4} + r_{r5} - r_{r6} - 2 * r_{9} - 2 * r_{10}$$

$$\frac{d\theta_{N_2}}{dt} = r_{r7} - r_{r8} - r_{d2} + r_{a2}$$

$$\frac{d\theta_{H_2}}{dt} = r_{r9} - r_{r10} - r_{d3} + r_{a3}$$

$$\frac{d\theta_{*}}{dt} = - \left(\frac{d\theta_{NH_3}}{dt} + \frac{d\theta_{NH_2}}{dt} + \frac{d\theta_{NH}}{dt} + \frac{d\theta_{N}}{dt} + \frac{d\theta_{H}}{dt} + \frac{d\theta_{N_2}}{dt} + \frac{d\theta_{H_2}}{dt} \right)$$

The initial parameter is input into the program by the command:

Y_{NH3}, Y_{N2}, Y_{H2}, θ_{NH_3}, θ_{N2}, θ_{H2}, θ_{NH_2}, θ_{NH}, θ_{N}, θ_{H}, θ_{*} = [a,0,0,0,0,0,0,1]

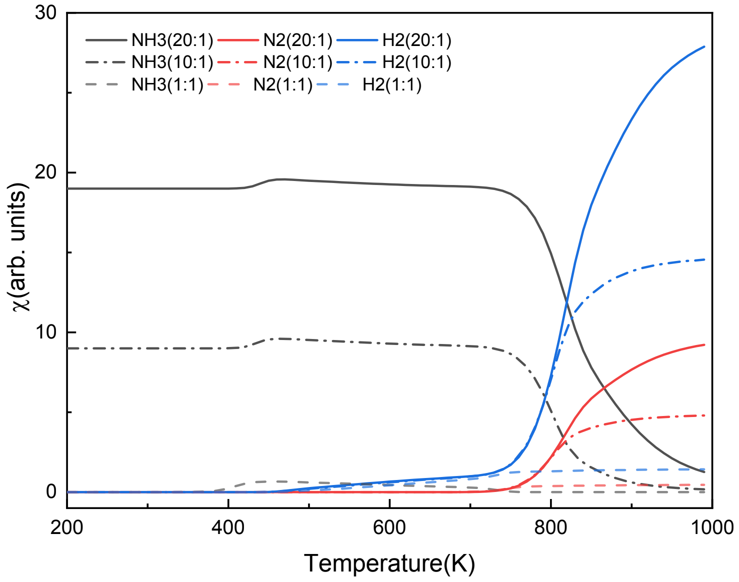

Figure S10. The reaction state ratio (χ) of NH_3, N_2, H_2 as functions of temperature on Ru(0001), Ru(111) and Ir(111) on batch reactor simulations. The initial simulated conditions are an NH_3 ratio of 1:1, 10:1 and 20:1 with a free surface. The reaction time is 600 s.

VI. PDOS of adsorption structure

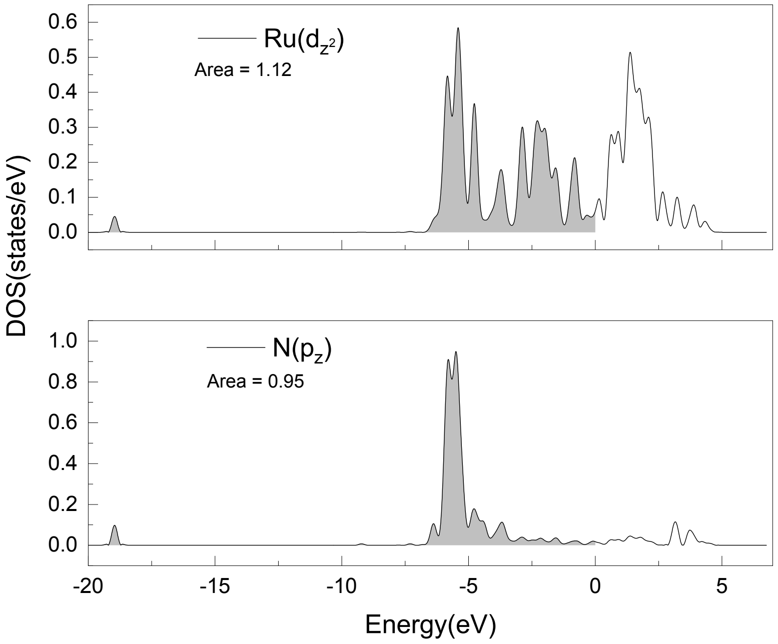

Figure S11. PDOS of N(p_z) and Ru(dz^2) upon NH_3 adsorption on Ru(0001).

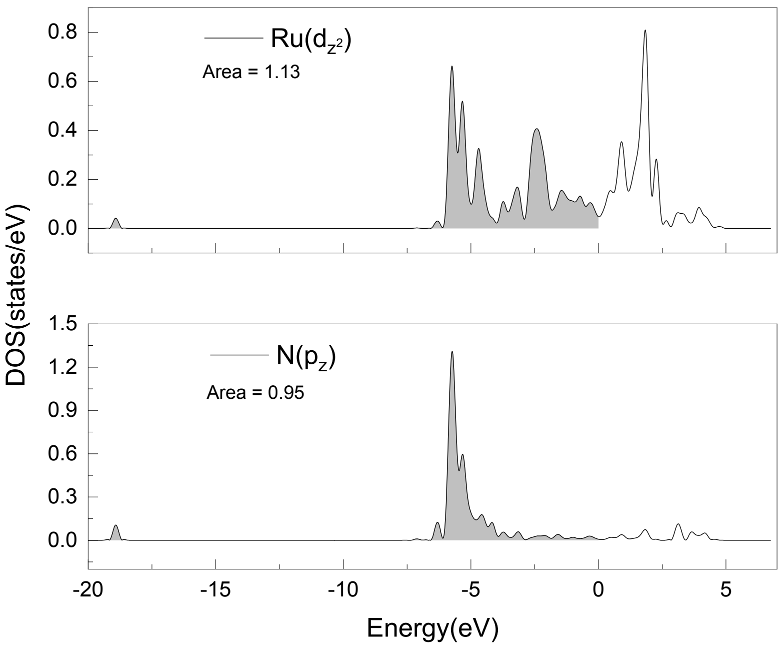

Figure S12. PDOS of N(p_z) and Ru(dz^2) upon NH_3 adsorption on Ru(111).

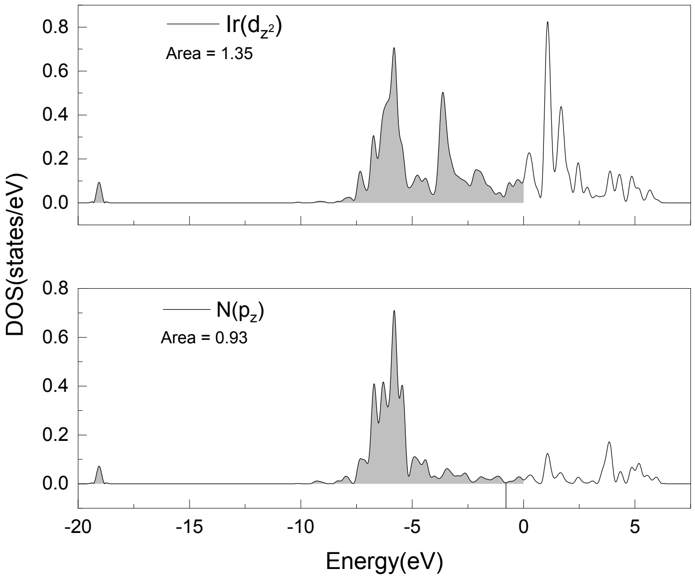

Figure S13. PDOS of N(p_z) and Ir(dz^2) upon NH_3 adsorption on Ir(111).

VII. Reference

(1) Chorkendorff, I.; Niemantsverdriet, J. W. *Concepts of Modern Catalysis and Kinetics*; John Wiley & Sons, 2017.

(2) Chase Jr, M. W.; Tables, N.-J. T. Data Reported in NIST Standard Reference Database 69,

June 2005 Release: NIST Chemistry WebBook. *J. Phys. Chem. Ref. Data, Monograph* **1998**, *9*, 1–1951.
(3) Ihsan, B. Thermochemical Data of Pure Substances. *and* **1995**, *934*, 587.
